# Caregiver-Centered Communication: A New Tool for Assessment of the Quality of Communication With Family Caregivers

**DOI:** 10.1093/geroni/igaa057.1891

**Published:** 2020-12-16

**Authors:** George Demiris

**Affiliations:** University of Pennsylvania, Philadelphia, Pennsylvania, United States

## Abstract

Improved communication with caregivers can lead to reduced caregiver anxiety and burden, improved quality of life, and better coping during a stressful time. Even though caregiver communication with the health care team is essential in gerontology, we are lacking standardized instruments to assess quality of communication. We describe the initial development and testing of the Caregiver Centered Communication Questionnaire (CCCQ). The questionnaire has 30 items with 5 subscales: exchange of information, fostering health relationships with team/provider, recognizing and responding to emotions, managing care and decision making. We conducted a cross-sectional survey of 115 family caregivers of older adults in home care and hospice. Cronbach’s α for the scale was 0.97. Internal consistencies of subscales were high, ranging from 0.82 to 0.93. Preliminary testing indicates the potential of CCCQ in assessing engagement and quality of communication; further testing is required.

